# Introduction of high-value *Crocus sativus* (saffron) cultivation in non-traditional regions of India through ecological modelling

**DOI:** 10.1038/s41598-022-15907-y

**Published:** 2022-07-13

**Authors:** Amit Kumar, Mamta Devi, Rakesh Kumar, Sanjay Kumar

**Affiliations:** CSIR-Institute of Himalayan Bioresource Technology, Council of Scientific and Industrial Research, Palampur, Himachal Pradesh 176 061 India

**Keywords:** Plant sciences, Ecology

## Abstract

*Crocus sativus* L. (saffron) is a globally used expensive spice. There are a few countries like Iran, Greece, Morocco, Spain, Italy, Turkey, France, Switzerland, Pakistan, China, Japan and Australia where this spice is cultivated and exported to other countries. India contributes 5% of the world's total production of which 90% is supplied only from its Jammu and Kashmir (J&K) regions. In India, the production of saffron from J&K is 3.83 tonnes whereas its annual demand is approximately 100 tonnes. In this country, there are geographical regions that have similar environmental and ecological conditions to J&K and possess the possibility of introducing this crop. Identification of such regions can be made using Ecological Niche Modelling (ENM). Therefore, 'MaxEnt' ENM was carried out using 103 environmental variables, 20 presence data and topographic parameters (elevation, slope and aspect) to find suitable regions for saffron production in unconventional areas of India. The achieved area under the curve for the model was 0.99. The precipitation and temperature were the main environmental variable influencing its cultivation. The saffron was sowed in these new modelled locations in India representing its various states such as Himachal Pradesh, Uttarakhand, Arunachal Pradesh, Sikkim, Manipur and Tamil Nadu. The quality, as well as yield of saffron produced in some of these regions, were evaluated and found at par with the saffron grown traditionally in India. Based on the promising results obtained in this work, we are expanding saffron cultivation to more modelled areas in India to meet our national demand.

## Introduction

*Crocus sativus* L. (saffron) or Golden spice or Red gold or ‘Kesar’ belongs to the family 'Iridaceae'^1^ and is one of the most expensive spices in the world^2^.Saffron has several medicinal properties such as aphrodisiac, antispasmodic, antimicrobial, antibacterial, antifungal, antiseptic, and antiinflammatory^3,4^. It has anticancer medicinal properties also, which has elevated its cost^5^. Its stigma is used in food, pharmaceutical, cosmetic, perfumery and textile dyes industries^6,7^ in various ways (mostly in dry form). Saffron cultivation is labour intensive requiring hand labour in the various stages of its cultivation. Its stigma needs to be carefully picked from its flower manually. Thus harvesting followed by processing requires trained work hands^8^. Therefore, saffron is costly due to its high labour requirement, low yield, careful handling and limited worldwide distribution. Saffron is mainly cultivated in Iran, India, Greece, Morocco, Spain, Italy, Turkey, France, Switzerland, Pakistan, China, Japan and Australia. Iran ranks first in its production and almost 80% of the world demand for saffron is met by Iran^9^. India contributed only 5% of the world's total production. 90% of the country's saffron is produced in Jammu and Kashmir (J&K) and here it is mainly confined to Pulwama and Budgam districts^10^. Saffron production in J&K has been reported to decrease year by year (Table S1). From 1997 to 2015 the decrease in area and productivity of saffron was observed by 83% and 72%, respectively^11^.

The environmental and topographic conditions for its growth and development vary between countries^12^. In Italy, it is grown from 650 to 1100 m AMSL with an average rainfall of 700 mm^13^. In La Mancha, Spain it is found at 610 m AMSL^14^, whereas in Sahr-Kord, Iran it is distributed at 2066 m AMSL^15^. Its average annual temperature range in the world goes from 5.9 to 18.6 °C and its rainfall range is between 420 and 1370 mm^16^. Temperate, semi-arid and arid areas are some of the ideal climatic conditions favouring saffron in the world^13^. In India, it is mainly grown in temperate climate condition^17^, where the soils are loose and well-drained. The clay calcareous soil to silty-clay soil^13^and sandy to sandy-loam soil^11^ are generally considered good for its growth. The high soil pH value produces a high yield of Saffron^18^. In Jammu and Kashmir, soil pH ranges from 6.3 to 8.3 while soil electrical conductivity range between 0.09 to 0.30 dms^−1^ for saffron^19–21^. For flower development optimum temperature range is 23–27 °C in summer and winter temperatures should not be less than − 15 to − 20 °C^22^. Therefore, it was observed that environmental conditions (temperature and precipitation)^23,24^ play a prime role in saffron growth and development. However, these conditions serve as a basis for finding the suitable regions for their cultivation but that needs to be further explored to understand specific requirements for a targeted region favouring their optimum growth. The supportive factors such as quality planting materials, cultivation techniques and methodology for corm production, rate of survival after sowing, etc., also play an important role in its proper growth and development.

To increase the saffron production in India with the appropriate quality, many efforts have been made, and it has been cultivated in new areas from time to time but has failed to result in an optimum yield and quality^25^. The reason may be that those areas might have been targeted by visual judgements. Here through an analysis of environmental conditions, it was possible to evaluate that those areas that appeared similar to areas where saffron is already cultivated were not suitable for the growth of saffron. In this condition the modelling for precisely finding out the suitable habitats for saffron cultivation becomes imperative and one of such solutions is Ecological Niche Modelling (ENM). The ENM refers to tools that correlate species occurrence with the underlying environmental conditions. Therefore, this study was done to identify new areas in India through ENM where there is a possibility of saffron cultivation. The quality of saffron produced in such regions was also monitored in the laboratory by their chemical evaluation. Our main intention is to introduce saffron in a large area to meet at least our domestic household and industrial demand. Our idea is that with our effort there will not only be an increase in the area under saffron cultivation but it will also generate employment and provide additional income to the farmers involved in its cultivation.

## Results and discussion

The ENM identified 06 states in India (a total 5.09% area of India) having the potential for cultivations of saffron. Of which 1.09% come under high suitability, 2.46% under medium suitability, and 2.34% under low suitability classes. Of the total 5.09% area in India, northwestern Himalaya had 4.81% area (consisting of 2.12% in Jammu & Kashmir, 1.41% in Uttarakhand); eastern Himalaya had 0.65% (0.62% in Arunachal Pradesh, 0.03% in Sikkim) and Tamil Nadu in Southern India had 0.44% area of India favourable for the growth of saffron.

**Figure 1:**
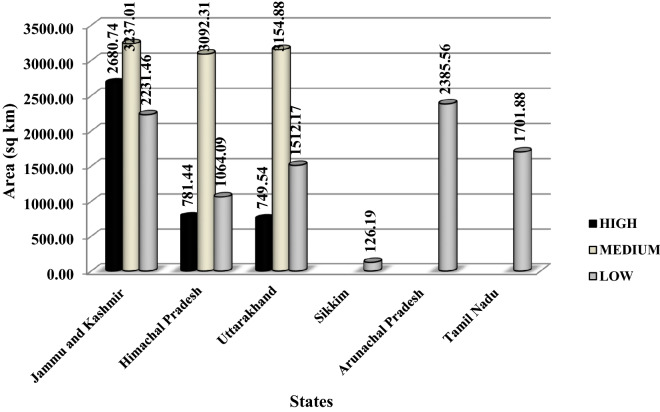
State-wise probable areas suitable for saffron cultivations in India.

The eastern Himalayan states and southern Indian states had only low suitability classes, whereas J&K, Himachal Pradesh and Uttarakhand consisted of all the 03 suitability classes, i.e., low, medium and high. Of the total 5.09% area identified as a probable niche area for saffron, J&K had the highest area under the high suitability class (4.91%) followed by Uttarakhand (1.52%) and Himachal Pradesh (1.34%) (Fig. 1).

To evaluate the sensitivity and performance of our model, the Area under the curve (AUC) was tested^26^, which displays the omission rate and predicted area at different thresholds. It ranged from 0.95 to 0.99 in our case (Table 1, Fig. S1). The AUC ranges from 0 to 1^27^ where AUC having 1.0 indicates perfect discrimination^28^ by these models. To avoid the ambiguity in the ENM model performance, the smoothing and calibration of datasets were also carried out^29^. The model run was tuned with 15 iterations for averaging the results by holding 30% of the data for testing purposes for model performance as a random test percentage. With these settings, we have calibrated the 7 different ENM model runs using different sets of environmental variables and presence data. These ENM provided 7 sets of modelled niche regions, their respective AUC values and predictive variables (Table 1). The higher values of site suitability indices obtained in the above ENMs were extracted and combined in a single merged output to tap the best possible niche habitats for saffron. The remaining indices values were discarded by way of visual judgement based on field experience as such lower indices represented the regions where the occurrence of saffron was logically not possible. The above-merged ENM output with high suitability indices was recalibrated with topographic variables such as elevation, slope and aspects. The regions, which did not meet the thresholds for the topographic parameters, were again discarded. Thus, we have obtained the final ENM result which was the combination of 7 ENM runs re-calibrated with topographic variables. To access its accuracy, the 12 presence data of saffron in India were overlaid on the above ENM result and it was observed that these locations were coinciding with modelled niche habitat.

**Table 1:** Comparative analyses of results of various ENM.

Method	Five top most important environmental variables	(AUC)
ENM I	Bio 11—Mean temperature of coldest quarter	0.956
	Bio 9—Mean temperature of the driest quarter	
	Bio 12—Annual precipitation	
	Bio 10—Mean temperature of warmest quarter	
	Bio 18—Precipitation of warmest quarter	
ENM II	Bio19—Precipitation of Coldest Quarter	0.985
	Solar Radiation—Solar Radiations (kJ m^−2^ day^−1^) of November month	
	Bio 11—Mean temperature of Coldest Quarter	
	Precipitation—December month precipitation (mm)	
	Bio 6—Min Temperature of Coldest Month	
ENM III	Precipitation—January month precipitation (mm)	0.986
	Maximum Temperature—Maximum temperature of November month (°C)	
	Maximum Temperature—Maximum temperature of December month (°C)	
	Precipitation—March month precipitation (mm)	
	Average Temperature—Average temperature of October month	
ENM IV	Bio19—Precipitation of Coldest Quarter	0.974
	Bio 11—Mean temperature of Coldest Quarter	
	Bio 3—Isothermality (BIO2/BIO7) (*100)	
	Bio 1 -Annual Mean Temperature	
	Bio 17 -Precipitation of Driest Quarter	
ENM V	Solar Radiation—Solar Radiations (kJ m^−2^ day^−1^) of November month	0.957
	Solar Radiation—January month Solar Radiations (kJ m^−2^ day^−1^)	
	Precipitation—December month precipitation (mm)	
	Bio19—Precipitation of Coldest Quarter	
	Bio 3—Isothermality (BIO2/BIO7) (*100)	
ENM VI	Bio19—Precipitation of Coldest Quarter	0.986
	Solar Radiation—Solar Radiations (kJ m^−2^ day^−1^) of November month	
	Precipitation—December month precipitation (mm)	
	Bio 3—Isothermality (BIO2/BIO7) (*100)	
	Solar Radiation—January month Solar Radiations (kJ m^−2^ day^−1^)	
ENM VII	Bio 17—Precipitation of Driest Quarter	0.999
	Wind Speed—Wind speed (m s^−1^) of August month	
	Wind Speed—Wind speed (m s^−1^) of October month	
	Precipitation—December month precipitation (mm)	
	Bio 14—Precipitation of Driest Month	

*AUC* area under curve.

Now a day, the ENM based on ensemble species distribution models like BIOMOD^30,31^ are being recommended, which combine several individual models for a unified niche habitat prediction. However, in this study model was calibrated with a different approach rather than using any ensemble model because of the complexity in the tuning of such a model. At the same time, the model handling is also lesser understood due to the lack of availability of enough supportive information. Moreover, we had fewer presence data for modelling and in many cases, the individual model has been reported to outperform the ensemble model^32^. Instead, the highest predictive classes were obtained by way of weighted overlay analysis in GIS environment of 7 ENM outputs obtained through different sets of environmental variables and saffron presence data.

Before the MaxEnt model run, the number of 103 environmental variables for ENM was restricted using the auto-correlation approach, which identifies and removes interrelated variables to avoid their repetitions. This was followed to avoid model complexity for its improved predictive capacity. We have used all possible environmental variables instead of restricting to those which are already known based on the biology of the occurrence of saffron. These are temperature, rainfall and elevation in the case of saffron as mentioned in the introduction section but these values are generic representing the global locations of its occurrences and may not be appropriate for ENM. Saffron is a complex crop whose ENM requirements vary for its various traits like its vegetative growth, number of flowering, the yield of its stigma and healthy corm production. Thus ENM statistical analyses helped us in retrieval of environmental variables specific to Indian regions required for optimum growth of saffron. Since availability of seamless spatial environmental local data was a constraint for ENM, the geo-referenced global coverage of ‘bioclim’ bioclimatic variables consisting of a wide range of environmental parameters were found appropriate for this purpose. The ENM analysis of predictive variables using Jackknifing of regularised training gain has suggested precipitation (during driest and coldest quarters) as the important environmental variable influencing saffron’s cultivation (Table 1). The total rainfall of 742.7 mm was the favourable environmental conditions for optimum growth of saffron^33^. The J&K in India receives sufficient precipitations during these periods producing favourable conditions for saffron. The importance of rainfall in saffron cultivation can be linked to its climatic condition during 2003–2004 when the yield of Saffron in J&K was observed low (1.57 kg) because of prevailing drought conditions^11^. Other important variables influencing saffron cultivation were identified as temperature and solar radiations during the coldest quarter. Temperature ranging from 20.9 to 27.8 °C with an average temperature of 28.2 °C was found suitable for the growth and flowering of saffron^33^.

The ENM provided modelled regions which are environmentally suitable for the cultivation of saffron. But the accurate identification of the location of cultivation in these regions is crucial for their appropriate growth and development, which includes the fertile sandy-loam texture of the soil and availability of irrigation, which should also be supported with saffron specific agro-practices^32^. Porous soil increase soil permeability helping in root growth, the irrigation keeps soil saturated especially during the driest and coldest rainfall conditions when the availability of water is low. The agro-practices like time of sowing and harvesting of saffron, application of growth-promoting media (fertilisers/microbes), spacing between plants and soil depth, etc., is required for accumulation of compounds in saffron for industrial needs, growth of the appropriate size of corms, flower production, and stigma yield. The ENM result was also overlaid on Survey of India topographic sheets and Google Earth online application to find out the approachability of suitable locations from the road and for other logistics requirements required for the cultivation and introduction of saffron at these new locations.

The identified locations were visited by our team of agronomists and six new locations in the north-western Himalayan region (Moorang in Kinnaur; Suppa and Sathli in Bharmour, Chamba; Langha and Palampur in Kangra of Himachal Pradeh and Kapkote in Bageshwar, Uttarakhand) two in eastern Himalayan regions (Shipgyer, North Sikkim and Imphal, Manipur) and one in southern India (Ooty, Tamil Nadu) were identified for the plantation of saffron. These locations are devoid of any history of saffron production. The cultivation of saffron was started at these new locations with the help of local farmers under the supervision of our scientists.

**Figure 2:**
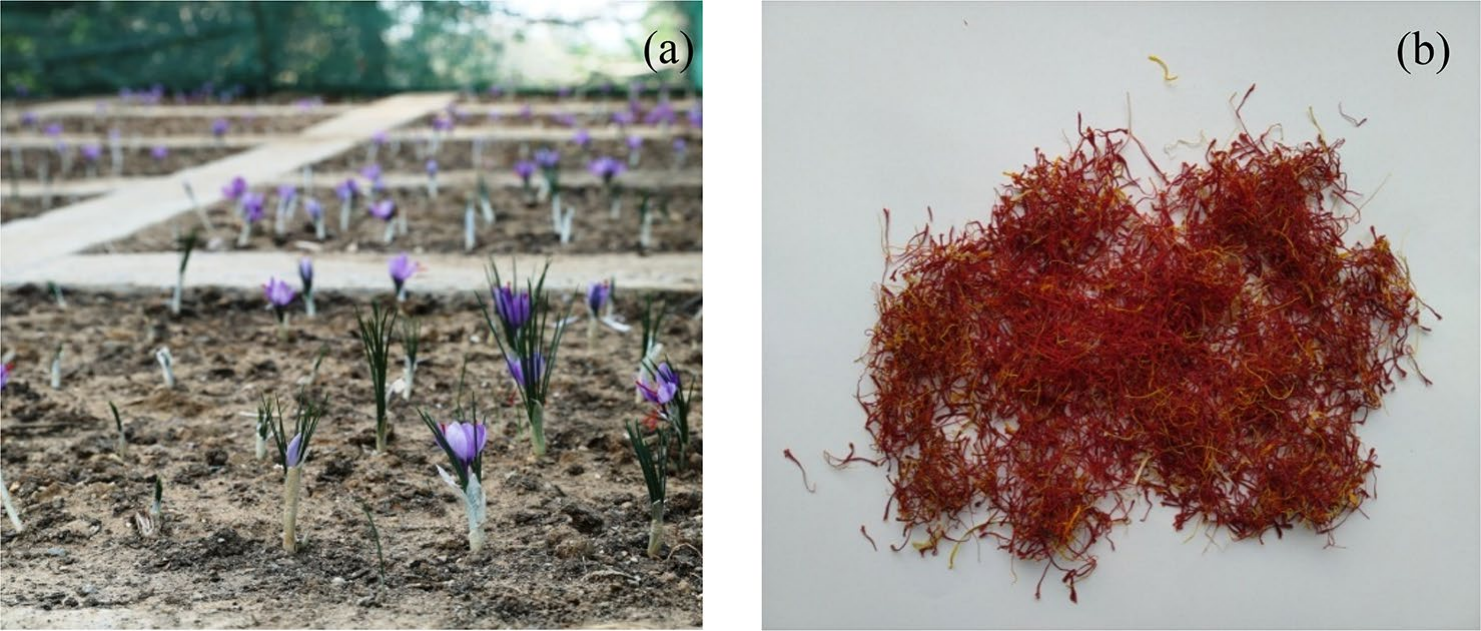
(**a**) Flowering in saffron, and (**b**) dried saffron stigmas.

Agronomical trials were conducted in new regions in the northwestern Himalaya maintaining similar agronomic management practices for the two years and it was found that the length of stigma (saffron) was found more or less similar (3.5–3.6 cm) at new cultivated locations (Fig. 2)﻿, which is at par with the reported average length of stigma from J&K, i.e., 2.5 cm^34,35^ and Iran, i.e., 3 cm^36,37^. The obtained yield was more in Bharmour (2.4 kg/ha) of Himachal Pradesh in the northwestern Himalayas which is at par with the national average yield (2.6 kg/ha). The yield obtained at other places ranged from 1.61 to 2.21 kg/ha. The two years’ average yield of saffron was found as 2.255 kg/ha, 2.005 kg/ha, and 1.665 kg/ha respectively at high, medium and low suitability regions. These statistics are based on 1-year cultivation data which need to be repeated in the second year for more accuracy. In a one-year trial the yield varied from 0.045 to 2.10 kg/ha with 3.5 cm stigma length in the eastern Himalayan and southern Indian regions.

Crocin and Safranal are the main compounds of saffron which are responsible for its colour and aroma, respectively. Concentrations of these compounds in their stigmas determine the quality and market values of saffron. The saffron from randomly selected 05 new locations from 02 states (Himachal Pradesh and Uttarakhand) as well as a traded sample of saffron in the J&K markets was chemically evaluated by the UPLC-MS method at our analytical laboratory. The Crocin and Safranel contents on these locations respectively ranged from 36.2 to 53.7 mg/g and 2.9 to 4.9 mg/g. The Crocin and Safranel contents in traded samples were 35 mg/g and 0.8 mg/g respectively. Thus,it was revealed that the Crocin and Safranal contents in the newly cultivated saffron were at par with the quality of traded saffron in India.

The accumulation of Crocin and Safranel, the length of stigma and the yield of the saffron varied from location to location. To have consistency in the above production, location-specific agro-techniques are required for saffron cultivation. The site-specific soil treatments with growth-promoting microbes or fertilisers, correct timings of sowing and harvesting of saffron, timely and optimum irrigation support, etc., are some of the measures which have been suggested under the agro-techniques. The post-harvest management of saffron is also an important parameter to maintain the quality of supply of saffron to the industries.

Presently, India’s saffron consumption is estimated at 20 tonnes a year, while the demand is 100 t/year, half of which is met by leading producers Iran, Spain and China (https://www.tpci.in/blogs/product-profile-saffron/). The area identified under the high suitability class for Saffron cultivation in India was 4211.72 km^2^, which is sufficient to meet the annual demand of India. The saffron production in India is 4 t/year, while its productivity is approx.3 kg/ha/year. Therefore, the area required for the cultivation of saffron to meet our annual demand can be calculated approximately as 333.33 km^2^. Looking at the above encouraging results of saffron introduction in India, we are now bringing more area under saffron cultivation in its suitable niche habitat identified through ENM. We are aiming to meet our country's demand for saffron with the help of networking with local and national agencies involved in the cultivation of these types of high-value crops.

## Conclusions

The study concluded that ENM using MaxEnt is an important and useful tool to introduce high-value crops like saffron in new areas where it was earlier not grown. This technology can also be used for other similar commercial crops. The ENM helped in identifying probable unconventional areas where we could introduce saffron cultivations. The increase in altitude, optimum temperature, lesser rainfall, moderate slope and aspects other than S, SW and W were found favourable for saffron cultivation. The deviations from the above influence its quality and yield. The optimum temperature is required for the increased number of flowers while rainfall influences saffron yield (stigma) and its biochemical parameters. The quality and quantity of saffron cultivated in those regions were observed at par with saffron grown in conventional areas in India and Iran. There is opportunity for the introduction of saffron in a larger area in India, which has been identified as climatically suitable for saffron through ENM. Saffron can be cultivated in these modelled areas without compromising on its quality and yield with the help of a region-specific agro-practices package developed by our agronomists. This will not only fulfil the country’s domestic need for saffron but the farmer will also be benefited economically because of the strong market and domestic demands of saffron resulting in an assured consumption of their produce.

## Study area

The present work was conducted to find out suitable habitats for the cultivation of saffron in India. It extends from 8° 4′ to 37° 6′ N latitude to 68° 7′ to 97° E longitude (Fig. S2). India is the seventh-largest country in the world.

The northern frontiers of India are defined largely by the Himalayan mountain range, where the country borders China, Bhutan and Nepal. The elevation ranges from sea level to approximately 8000 m AMSL. Most of the peaks are situated in the Sikkim and Uttarakhand state of northern India. The highest mountain peak is Kangchenjunga (8586 m) in India and ranked the highest summit in the world it is located at the border of India and Nepal in the great Himalayas range in Sikkim. In the far northeast, the Chin Hills and Kachin Hills are covered with the forested mountainous region, separating India from Myanmar. On the east, its border with Bangladesh is largely defined by the Khasi Hills, Mizo Hils, and Indo-Gangetic Plain. Its western border lies with Pakistan. Southern Indian boundary is covered by the Arabian Sea, southwest there is Lakshadweep Sea, and the Bay of Bengal on the east. Because of this large coverage, there are a lot of variations in its physical appearance, climatic condition, rainfall, altitude, soil type, ecology, etc., leading to the possibility of the introduction of saffron in its new areas for its cultivation.

## Materials and methodology

Among various ENM algorithm methods, the MaxEnt statistical model is supposed to be one of the best models to predict the niche habitats of plants using their location of occurance^38–46^. MaxEnt technique compares a set of recorded locations of particular samples^38,47^ with relevant environmental variables to create an environmental profile that is afterwards located in another geographic region. One of the important features of the MaxEnt model is that it can be run with presence-only data, as the absence data are generally not available^38,39,48^.

**Figure 3:**
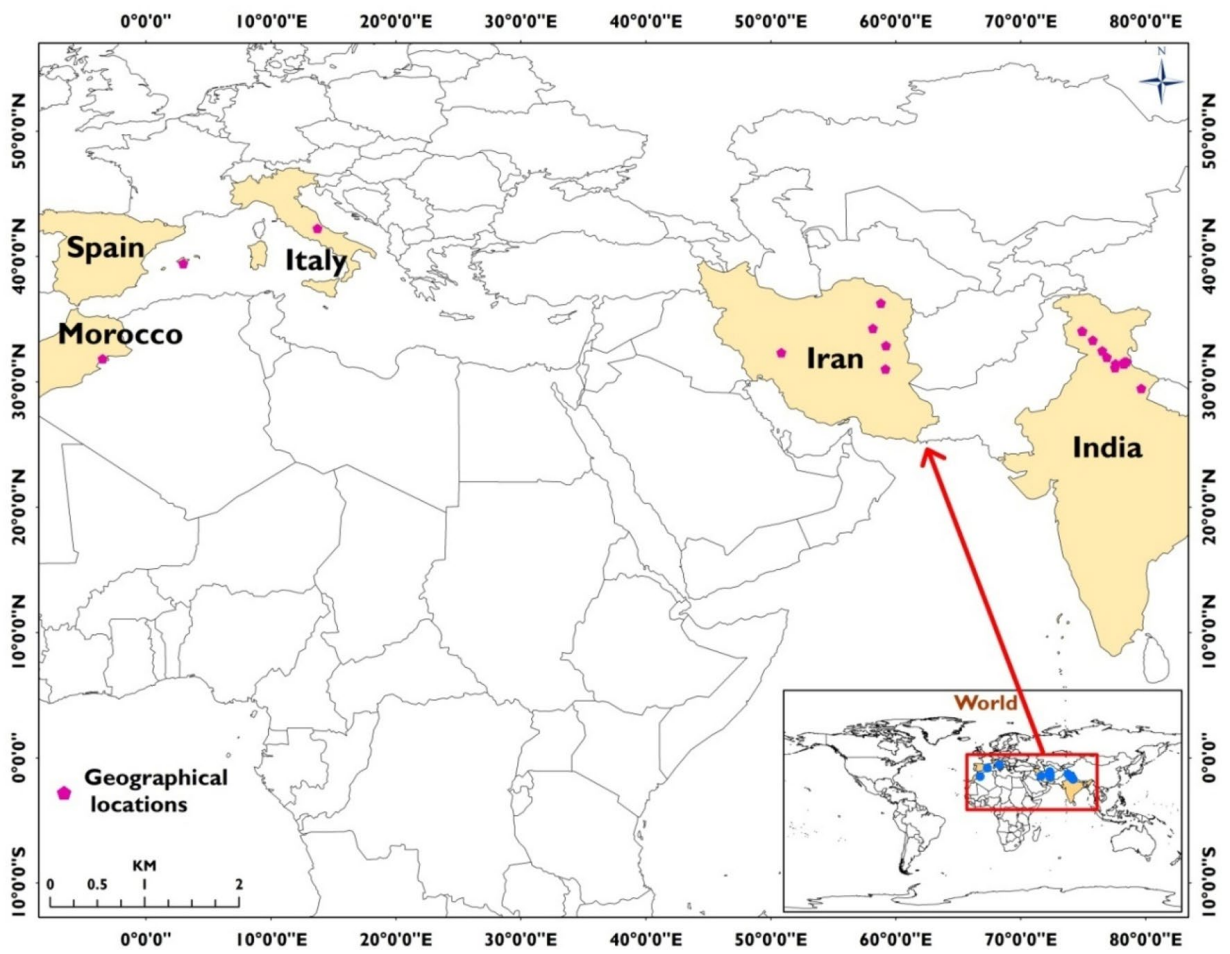
Geographical locations of *Crocus sativus* L. (saffron) used for ENM (prepared using ArcGIS 10.4.1; https://www.esri.com/).

Presence data of occurrence of saffron are one the important parameters for ENM modelling. Thus, surveys were conducted to record geographical coordinates of locations where saffron is traditionally grown using a handheld GPS device. A total of 12 such locations were recorded from Jammu and Kashmir union territory, Himachal Pradesh and Uttarakhand states of India. Saffron coordinates were also retrieved from the Global Biodiversity Information Facility (GBIF) web portal (https://www.gbif.org/what-is-gbif). From this site, 449 locations of saffron cultivations were identified but 30% of them were observed as preserved specimens stored at locations other than its cultivation. Some of the other locations, when plotted on google earth, represented built-up features and catchments of water bodies, and thus were not found appropriate as saffron locations. Also, more than 6 species of saffron are reported from Iran itself which are similar to saffron (*Crocus sativus*) and thus identification of its correct species for ENM was also a major concern from GBIF datasets^49,50^. Therefore, we have used coordinates of only those locations, which could be cross-checked from literatures in which they have mentioned the presence of *Crocus sativus* at such locations. Finally, 8 such authentic coordinates from Iran (n = 5), Italy (n = 1), Spain (n = 1), and Morocco (n = 1) were identified for the ENM. Thus, altogether 20 geographical points were used for the ENM (Fig. 3), which are optimum for ENM run for species like saffron which has a narrow occurrence^51^.

The second important factor for ENM is its environmental variables, which were downloaded from the WorldClim site (http://www.worldclim.org). The global climate version 2 worldClim data consisting of 103 variables were used for ENM (Tables 2, 3). Among these, the 19 environmental layers (Bio 1–Bio 19) are mainly related to averaged temperature and precipitation from 1970 to 2000. The rest 84 variables are the combination of monthly temperature, precipitations, solar radiations and wind speed from 1970 to 2000. These bioclimatic variables represent annual trends (e.g., mean annual temperature, annual precipitation), seasonality (e.g., annual range in temperature of the coldest and warmest month), and precipitation of the wet and dry quarters). A quarter is three months (1/4 of the year). The details of these environmental variables have been provided in Tables 2 and 3. For further refinement of the ENM results, the terrain data such as slope, aspect and elevation were used which were prepared from the Space shuttle radar topography mission (SRTM) Digital Elevation Model (DEM) having 30 m resolution.

**Table 2:** Mean environmental variables (1970–2000).

Bio 1	Annual mean temperature
Bio 2	Mean diurnal range (mean of monthly (max temp–min temp))
Bio 3	Isothermality (BIO2/BIO7) (*100)
Bio 4	Temperature seasonality (standard deviation*100)
Bio5	Max temperature of warmest month
Bio 6	Min temperature of coldest month
Bio 7	Temperature annual range (BIO5–BIO6)
Bio 8	Mean temperature of wettest quarter
Bio 9	Mean temperature of driest quarter
Bio 10	Mean temperature of warmest quarter
Bio 11	Mean temperature of coldest quarter
Bio 12	Annual precipitation
Bio 13	Precipitation of wettest month
Bio 14	Precipitation of driest month
Bio 15	Precipitation seasonality (coefficient of variation)
Bio 16	Precipitation of wettest quarter
Bio 17	Precipitation of driest quarter
Bio 18	Precipitation of warmest quarter
Bio 19	Precipitation of coldest quarter

**Table 3:** Mean monthly environmental variables (1970–2000).

1	Minimum temp. (°C) [Monthly Avg. of 1970–2000] = 12 variables
2	Maximum temp. (°C) [Monthly Avg. of 1970–2000] = 12 variables
3	Average temp. (°C) [Monthly Avg. of 1970–2000] = 12 variables
4	Precipitation (mm) [Monthly Avg. of 1970–2000] = 12 variables
5	Wind speed (m s^-1^) [Monthly Avg. of 1970–2000] = 12 variables
6	Water vapour pressure (kPa) [Monthly Avg. of 1970–2000] = 12 variables
7	Solar Radiations (kJ m^-2^ day^-1^) [Monthly Avg. of 1970–2000] = 12 variables
Total 84 variables	

**Figure 4:**
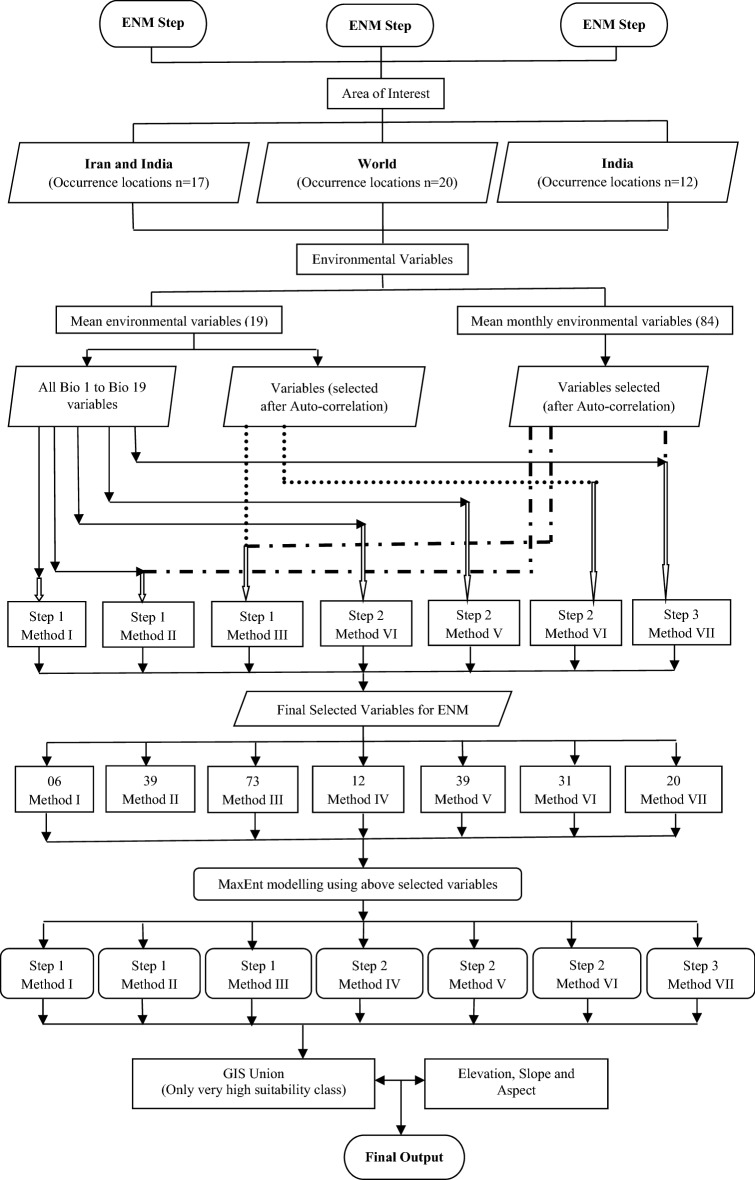
Flow chart of the methodology followed in the study.

To find out modelled probable niche areas of saffron in India, the ENM was carried out following 03 steps consisting of seven methodologies (Fig. 4). In the first method of step one, 19 bioclimatic variables (Bio 1–Bio 19) were considered for the model run. After, statistical auto-correlation, only six among 19 was used for the model run. In the second method also, in addition to these 19 variables (Table 2), 20 selected variables from 84 variables (Table 3) were considered after auto-correlation. In the third method, all the 103 variables were considered and among these, the model was run using 73 variables selected after auto-correlation. The above one, two and three ENM were run using 17 presence locations (step 1), 12 from India and 7 from its neighbouring country Iran (Fig. 4). Step two, methodologies include IV, V, and VI (Fig. 4) where all the 20 geographical locations of occurrences of saffron in the world were used for the MaxEnt run. In method IV, among bio 1 to bio 19, only 12 environmental variables were selected for the model run. In method V, a total of 39 variables were used, 19 from bio 1 to bio 19 (Table 2) and 20 selected from 84 variables (Table 3). A total of 31 environmental variables were used for modelling in method VI, which were retrieved from all 103 variables after the auto-correlation process (Fig. 4).In step three (method VII), a total of 39 environmental variables were used for 12 locations in India only (Fig. 4). These variables are all 19 variables (bio 1 to bio 19) plus 20 selected from the other 84 variables.

**Figure 5:**
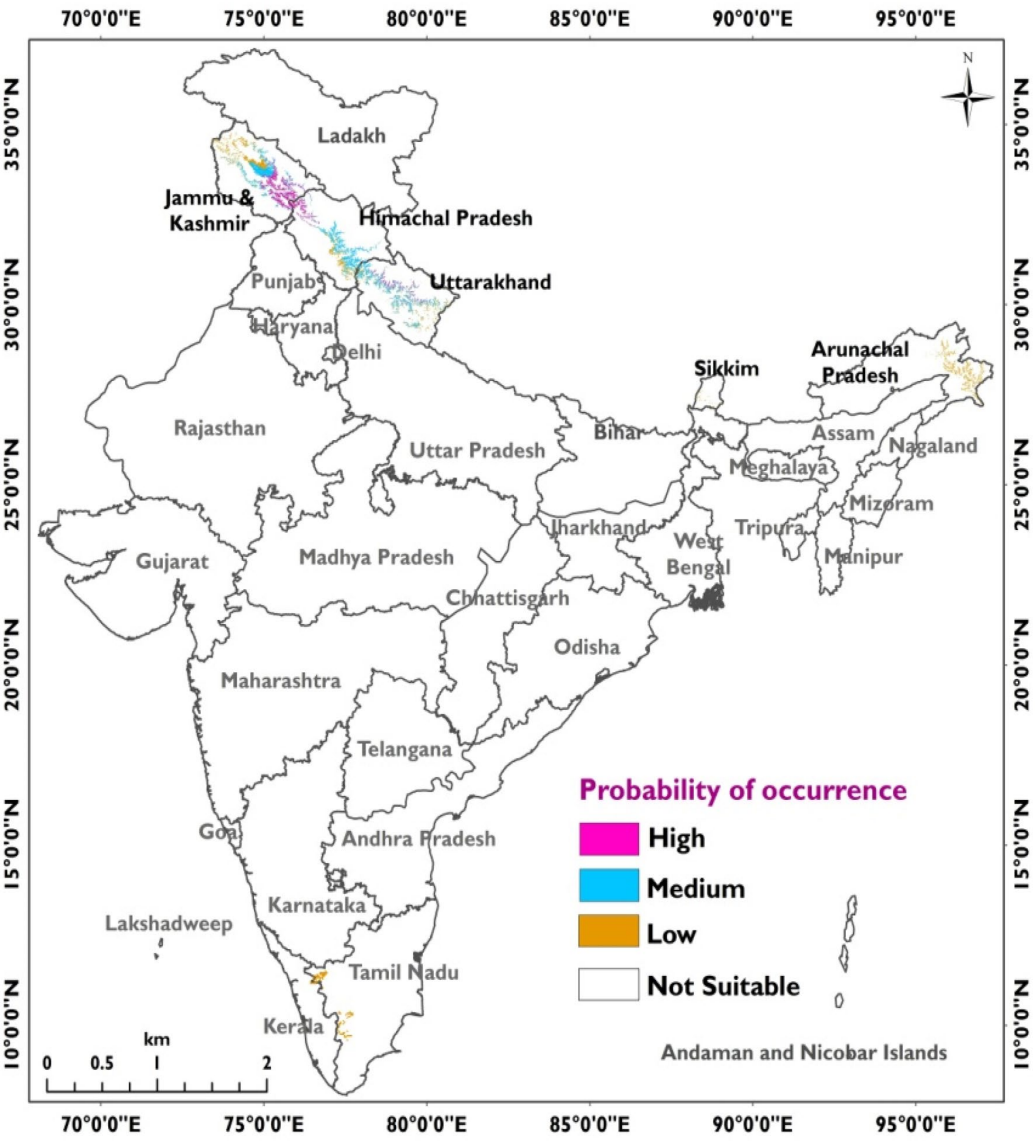
Modelled probable niche areas of *Crocus sativus* (Saffron) in India (prepared using ArcGIS 10.4.1; https://www.esri.com/).

We have followed 3 steps and 7 methodologies, which resulted in 7 separate MaxEnt outputs. These outputs had a range of site suitability indices associated with them which were divided into four suitability classes, i.e., low, medium, high and very high. Among these four categories, very high suitability classes were again picked from all the above 7 ENM outputs and were later merged in the Geographic Information System (GIS) environment as a single merged output**.** Before this, the regions having very high suitability classes in each of these 7 ENM outputs were assigned a weight of ‘1’ and rest of the classes were assigned ‘0’ weights. The merged output thus had very high suitability classes having cumulative weights that ranged from 1 to 7. Areas having elevations lesser than 1450 m and more than 2860 m in Northern India and areas having more than 610 m elevations in Southern India were excluded from the above output (Fig. 4). In the next step, regions having slopes other than gentle to moderate and aspects other than S, SW and W were also removed from the above output. These criteria for elevation, slope and aspect were finalized by overlaying geographical coordinates of 12 locations in India over SRTM 30 m DEM, where saffron is cultivated. Now at the end, the site suitability weights associated with the above output were finally grouped into 03 categories such as high suitability class (cumulative weight = 4–7), medium suitability class (cumulative weight = 2–4) and low suitability class (cumulative weight = 1), to arrive at a final map depicting probable niche areas for cultivations of saffron in India (Fig. 5).

## Supplementary Information

Supplementary Information.
