# Supplementary material for: Introduction of high-value Crocus sativus (saffron) cultivation in non-traditional regions of India through ecological modelling
Source: Sci Rep. 2022 Jul 13;12:11925. doi: 10.1038/s41598-022-15907-y (PMC9279281; doi:10.1038/s41598-022-15907-y)
Supplement: Supplementary file 1 — Supplementary Information. [file 41598_2022_15907_MOESM1_ESM.docx]

**Introduction of high-value *Crocus sativus* (saffron) cultivation in non-traditional regions of India through ecological modelling**

**Supplementary Materials**

**Tables**

| **Year** | **Area (ha)** | **Production (t)** | **Yield (kg ha-1)** |
| --- | --- | --- | --- |
| 1997 | 5707 | 15.95 | 2.8 |
| 1998 | 4161 | 12.88 | 3.13 |
| 1999 | 2880 | 7.65 | 2.27 |
| 2000 | 2742 | 3.59 | 1.88 |
| 2001 | 3075 | 0.30 | 1.57 |
| 2002 | 2989 | 6.50 | 2.96 |
| 2003 | 2928 | 5.15 | 1.66 |
| 2004 | 2436 | 6.86 | 3.75 |
| 2005 | 3110 | 7.04 | 1.63 |
| 2006 | 3130 | 6.50 | 2.25 |
| 2007 | 3010 | 8.20 | 2.15 |
| 2008 | 3000 | 7.70 | 2.50 |
| 2009 | 3280 | 9.46 | 2.34 |
| 2010 | 3785 | 9.55 | 2.50 |
| 2011 | 3790 | 9.85 | 2.52 |
| 2012 | 3674 | 10.00 | 2.72 |
| 2013 | 3674 | 11.50 | 3.13 |
| 2014 | 3674 | 15.00 | 4.08 |
| 2015 | 3674 | 9.60 | 2.61 |

Source: Jammu and Kashmir Agriculture Department and Jammu and Kashmir ENVIS

**Table S1**: Trend of production of saffron in Kashmir


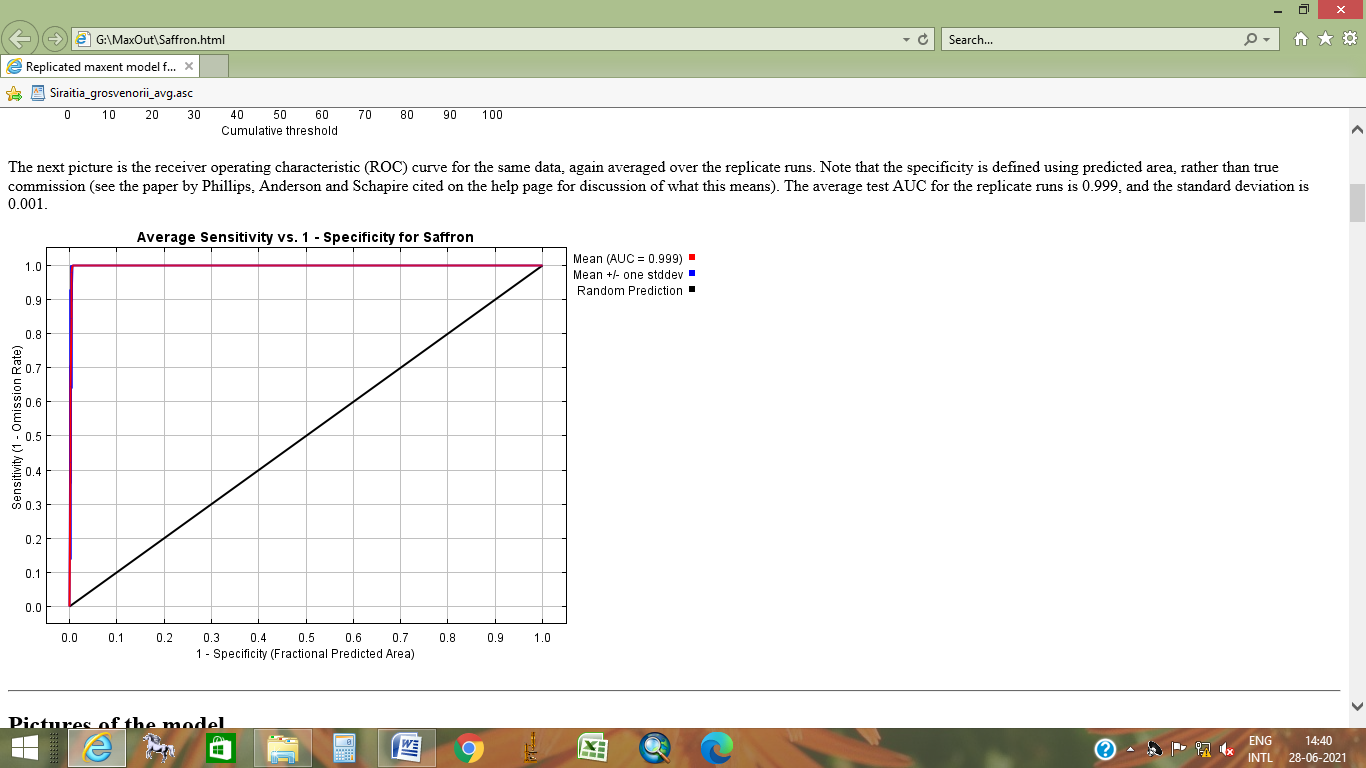

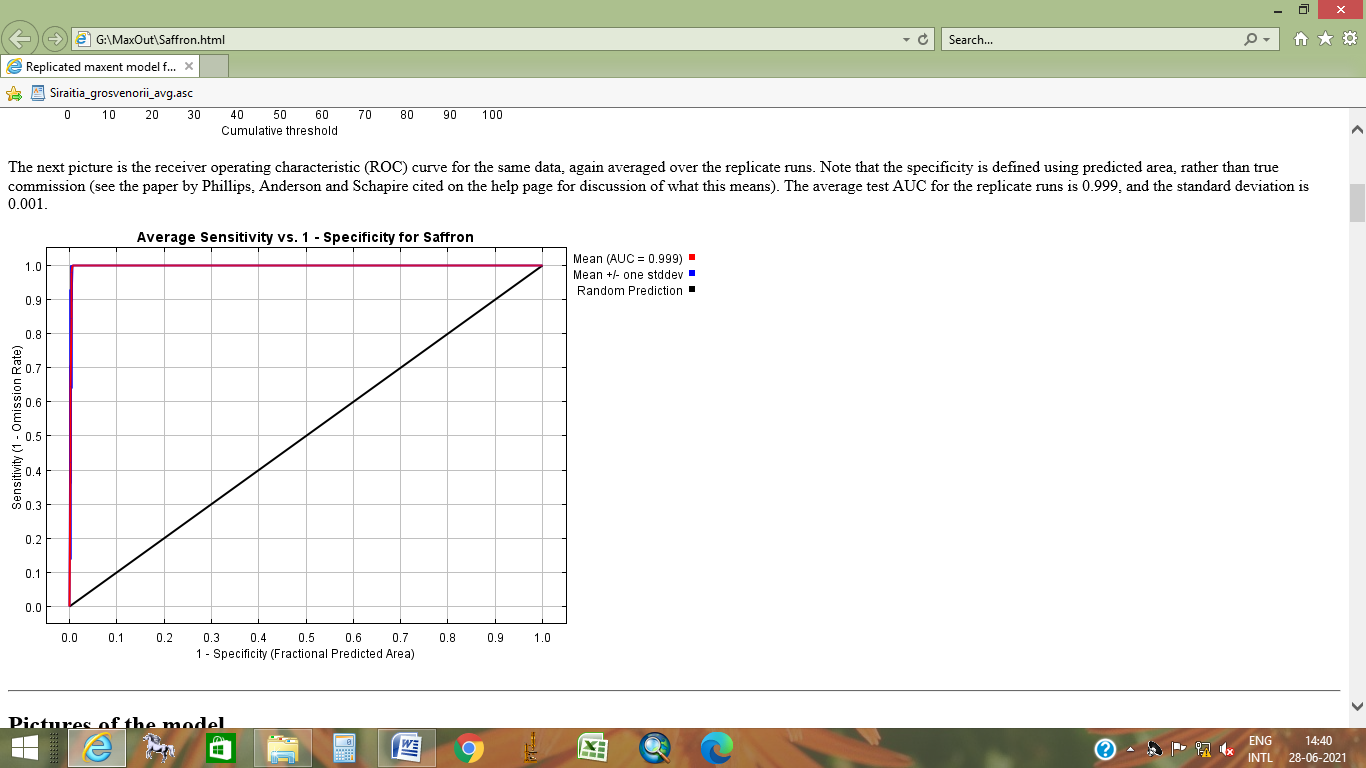


**Figure S1.** Area Under Curve (AUC) for evaluation of the performance of the model


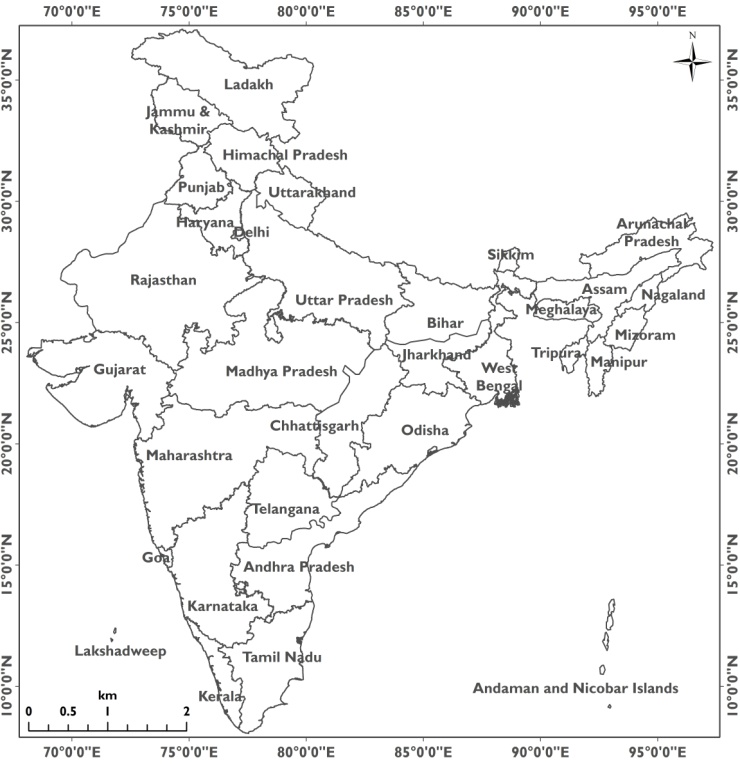


**Figure S2.** Study area (prepared using ArcGIS 10.4.1; https://www.esri.com/)
